# Evaluation and Characterization of High-Uniformity SiN_x_ Thin Film with Controllable Refractive Index by Home-Made Cat-CVD Based on Orthogonal Experiments

**DOI:** 10.3390/molecules30051091

**Published:** 2025-02-27

**Authors:** Caifang Li, Minghui Li, Jinsong Shi, Haibin Huang, Zhimei Li

**Affiliations:** 1 Key Laboratory of Jiangxi Province for Environment and Energy Catalysis, School of Chemistry and Chemical Engineering, Nanchang University, Nanchang 330031, China; 2 Institute of Applied Chemistry, Jiangxi Academy of Sciences, Nanchang 330096, China; 3 Jiangxi HAC General Semiconductor Technology Co., Ltd., Jiujiang 332020, China; 4 Jiangsu HAC AI-Machine Co., Ltd., Nantong 226300, China

**Keywords:** SiN_x_ thin film, Cat-CVD, orthogonal experiment, refractive index

## Abstract

Silicon nitride (SiN_x_) thin film is a promising coating with great physiochemical and optical properties. However, the preparation of films with good comprehensive properties still faces challenges. This study focused on developing a method for the preparation of uniform SiN_x_ thin film with a controllable refractive index using home-made catalytic chemical vapor deposition (Cat-CVD) equipment. Orthogonal experimental design was employed to investigate the effects of four key influence factors, including reaction pressure, the ratio of SiH_4_ to NH_3_, the ratio of SiH_4_ to H_2_, and substrate temperature. The response parameters evaluated were the refractive index, extinction coefficient, uniformity, and deposition rate of SiN_x_ thin film. Compared with the single-factor variable tests, an orthogonal experiment could obtain the optimal preparation process of the SiN_x_ thin film with the best comprehensive quality through the least number of experiments. At the same time, the microstructures of SiN_x_ thin film were analyzed by various characterization methods, including Fourier-transform infrared spectroscopy (FTIR), X-ray photoelectron spectroscopy (XPS), and scanning electron microscopy (SEM), to research the relationship between preparation factors and the properties of SiN_x_ thin film. This paper provides the theoretical guidance for fine-regulating the properties of SiNx thin film in practical applications.

## 1. Introduction

Silicon nitride (SiN_x_) is a functional material with excellent physiochemical properties, such as high hardness [1], high transparency [2], chemical stability [3], and a tunable optical band gap [4] and refractive index [5]. It has gained considerable attention due to its applications in photoelectronics, integrated circuits, and solar cells [6,7,8]. Specifically, high-quality SiN_x_ thin films on the surfaces of polycrystalline silicon solar cells can not only act as anti-reflection films but also play a key role in surface passivation, so as to significantly enhance the conversion efficiency of solar cells. For example, Elkady et al. [9] focused on the optimization parameters of plasma-enhanced chemical vapor deposition (PECVD) SiN_x_ for monocrystalline-silicon-solar-cell anti-reflection coating using pure SiH_4_ and NH_3_, which significantly enhanced the efficiency of solar cells by 1.23 percentage points. As it is an anti-reflective layer, the refractive index and extinction coefficient of thin film are essential parameters that directly affect the absorption rate of solar cells [10,11]. For SiN_x_ thin film, the anti-reflective effect is best to maximize optical transmission when the refractive index is 2.0 [12,13]. According to a large number of studies [14,15,16], there are several crucial effect factors affecting the performance of SiN_x_ thin film, including but not limited to the source gas ratio, reaction pressure, and diluent gas ratio. Guler [17] controlled the refractive index of SiN_x_ thin film through adjusting the flow rate of SiH_4_ and NH_3_ by PECVD and finally obtained SiN_x_ with a refractive index of 1.91. Canar et al. [18] obtained wide ranges of refractive indexes of 1.58–1.85 for SiO_x_N_y_ and 1.96–3.02 for SiN_x_, which were obtained by changing the flow rate of process gases. Therefore, it is a hot topic to change the conditions of the preparation of SiN_x_ thin film to regulate its properties such as its refractive index and extinction coefficient.

Up to now, the main methods for preparing SiN_x_ films have been physical vapor deposition (PVD), atomic layer deposition (ALD), and chemical vapor deposition (CVD). Among them, radio-frequency magnetron sputtering (RF-MS) is the representative technology of PVD, and this can prepare SiN_x_ films at room temperature, but due to the limitation of its technical principle, it prepares SiN_x_ films with slow deposition rates and poor uniformity [19]. ALD is known for atomic-scale accurate control and can prepare uniform SiN_x_ films with good step coverage. But it also has high requirements for equipment precision, which results in increasing costs and extremely low deposition rate. These drawbacks have limited its wide application [20]. LPCVD can deposit SiN_x_ films close to stoichiometric (N/Si = 1.33) but with strict conditions (high temperature and low pressure) and slow deposition rates [21]. PECVD is the most mature technology in the market, being widely used and researched. It can rapidly deposit SiN_x_ films at low temperatures on a large scale. However, there also exists an obvious defect of irreversible plasma damage, which will greatly affect the properties of the films [22,23]. Cat-CVD is a low-cost and high-efficiency deposition technology that can not only prepare uniform SiN_x_ films at low temperatures and low pressures but also maintain good step coverage and fast deposition rates [22]. Table 1 compare the parameters and characteristics of SiN_x_ films prepared by different techniques. Rai et al. [24] fabricated a-Si/SiN_x_/a-Si heterostructure SiN_x_ layers at 250 °C via Cat-CVD. Koichi et al. [25] prepared SiN_x_/i-a-Si stack films on Si substrates that possessed excellent passivation via Cat-CVD. The deposition rates of a-Si and SiN_x_ thin films were about 0.5 and 34 nm/min, respectively, and the refractive index of the SiN_x_ thin films was 2.00. Matsumura [26,27,28] proposed the reaction mechanism of a SiH_4_/NH_3_ system in Cat-CVD. In a word, SiH_4_, NH_3_, and H_2_ were decomposed to free radicals on a heated wire and the free radical underwent recombination to a Si-N or Si-Si deposit on a substrate to form a thin film [28]. H_2_ can be used as a reaction gas to promote catalytic decomposition and may also be regenerated as a reaction product [29]. Some of the possible reactions are as follows:

$$\begin{array}{l} {SiH}_{4}\to \cdot {SiH}_{3}+\cdot H\\ {NH}_{3}\to \cdot {NH}_{2}+\cdot H\\ H_{2}\to \cdot H+\cdot H\\ \cdot H+{NH}_{3}\to \cdot {NH}_{2}+H_{2}(↑)\\ \cdot {SiH}_{3}+\cdot {NH}_{2}\to H_{2}\;N-{SiH}_{3}\\ H_{2}\;N-{SiH}_{3}+\cdot {NH}_{2}\to H_{2}\;N-{SiH}_{2}-{NH}_{2}+H_{2}(↑)\\ \ldots \end{array}$$

Home-made Cat-CVD equipment cannot only enhance the equipment by film performance in time but also optimize the process of preparing films for different application scenarios, which undoubtedly facilitates the researching of equipment and film properties at a lower cost. Unfortunately, although Cat-CVD was developed decades ago, there have been very few research reports on it (less than 10% of the number of reports on PECVD, according to Google Scholar and Web of Science), and the investigation on it is still in its infancy.

In practical applications, SiN_x_ thin film as a solar cell anti-reflection film usually requires excellent comprehensive quality. However, the factors that affect its various properties may not be the same, which increases the workload of study. In addition, the preparation of SiN_x_ thin film is faced with challenges such as high equipment requirements, low deposition efficiency, and difficult-to-control film properties. Orthogonal experiment design is a powerful method for exploring the effects of multi-factor and multi-level experiments on response parameters with the least numbers of experiments [30]. This method was first proposed by Genichi Taguchi, and it can obtain the influence law of process parameters on response factors and the ranking of influence weights, and it has been applied to the performance analysis and optimization of SiN_x_ thin films [31,32].

In this work, we employed orthogonal experiment design to evaluate and optimize the preparation of SiN_x_ thin films via home-made Cat-CVD equipment. The effects of reaction pressure, the ratios of SiH_4_ to NH_3_ and SiH_4_ to H_2_, and the substrate temperature on the properties of SiN_x_ thin films were systematically investigated. The response parameters evaluated included the refractive index, extinction coefficient, uniformity, and deposition rate. This method can be combined with optimization and evaluation to obtain excellent-quality SiN_x_ thin films and optimal preparation conditions. The relationship between influence factors and the properties of SiN_x_ thin films were analyzed and discussed via several characterization techniques.

**Table 1:** Comparison of the relative parameters, advantages, and disadvantages of different methods for preparing SiN_x_ thin film.

Method	Preparation Parameters	SiNx Thin Film Parameter	Advantages (√) and Disadvantages (×)
RF-MS [19]	100 W, 1 Pa, Ar 80 sccm, N_2_ 5 sccm, Si target, 30 min	Refractive index: 1.4–2.1; Extinction coefficient: 0.01–0.5; Deposition rate: 0.06 nm/s	√ Low temperature; √ Without H pollution; × Low deposition rate; × Poor uniformity; × Low rate of utilization of target material.
ALD [33]	400 °C, 10 Torr, Bis(tertbutylamino)silane, N_2_ plasma 100 sccm, Ar 25 sccm,	N/Si: ~1.4, containing O and H; Refractive index: ~1.96; Density: ~2.9 g/cm^3^	√ High step coverage rate; √ Low deposition temperature; √ Atomic-scale thickness control; × Low deposition rate; × High cost.
LPCVD [21]	20 Pa, 830 °C, SiH_2_Cl_2_ 45 sccm, NH_3_ 180 sccm, 1620 s	N/Si: ~1.1; Density: 2.76 g/cm^3^; Deposition rate: 0.06 nm/s	√ SiN_x_ film has wide spectral range; √ Close to stoichiometric Si_3_N_4_; √ Without H pollution; × Low deposition rate; × High temperature (800–1200 °C).
PECVD [21]	RF-power of 100 W for 2.2s, 113 Pa, 300 °C, SiH_4_ 30 sccm, NH_3_ 30 sccm, N_2_ 1470 sccm, 120 s	N/Si: ~1.1; containing O and H; Density: 2.20 g/cm^3^; Deposition rate: 0.83 nm/s	√ Fast deposition rate; √ Low temperature; × High H content; × Irreversible plasma damage; × High cost.
Cat-CVD (This work)	3 Pa, 60 °C, SiH_4_:NH_3_ = 1:20, SiH_4_:H_2_ = 1:5, 480 s	N/Si: ~0.75, containing O and H; Refractive index: 2.026; Extinction coefficient: 0.056; Uniformity: 2.97%; Deposition rate: 0.3 nm/s	√ Fast deposition rate; √ Good uniformity; √ Low temperature; √ Low cost; × Hot wire life requires frequent maintenance and replacement.

## 2. Experimental Section

### 2.1. The Home-Made Cat-CVD Equipment

**Figure 1:**
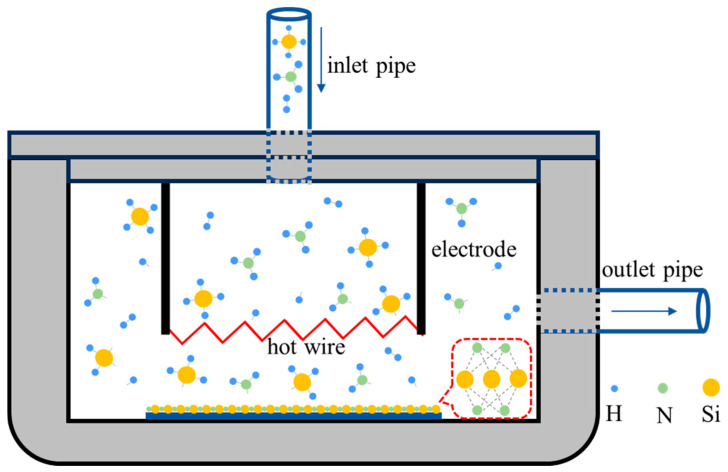
Schematic diagram of preparation of SiN_x_ film on glass substrate by Cat-CVD with SiH_4,_ and NH_3_ and/or H_2_.

Cat-CVD equipment mainly includes vacuum system, vacuum chamber, heating and cooling system, air source and air supply system, power supply and control system, and other structures. The working schematic diagram of Cat-CVD equipment is shown in Figure 1. Firstly, the pressure in vacuum chamber should reach 5 × 10^−4^ Pa (which is close to vacuum state) after the substrate is added to the base of chamber. The goal of this step is to remove the impurities as far as possible from the vacuum chamber. The hot wire is preheated to pyrolysis temperature in advance. At this time, the source gases begin to be injected from inlet pipe and can be decomposed on the hot wire in time. It should be noted that the source gases are injected continuously, so when the pressure in the vacuum chamber reach the target pressure, it is necessary to release the unreacted substances from outlet pipe. In this process, we need to control the flow rate of the inlet and outlet air so that the reaction pressure can keep a steady state.

### 2.2. Orthogonal Experiment Design for Preparing SiN_x_ Thin Film

Circle glass substrate (r = 100 mm) was washed with alcohol and ultra-pure water to make its surface clean. The films were deposited by pyrolysis of silane (SiH_4_) and ammonia (NH_3_) and/or hydrogen (H_2_). The deposition time was 480 s. All the SiNx film preparation experiments were carried out using the above-mentioned home-made Cat-CVD equipment (Jiangxi HAC General Semiconductor Technology Co., Jiujiang, China). In this work, the reaction pressure (P), ratio of SiH_4_ to NH_3_ (SiH_4_:NH_3_), ratio of SiH_4_ to H_2_ (SiH_4_:H_2_), and the substrate temperature (T_s_) were investigated. Level of an influence factor refers to the specific value or state of a factor in an experiment. Each factor can have multiple levels, and these levels are usually pre-set to study the effect of that factor on the experimental results. Considering the balance between home-made equipment limited conditions and result representativity, the level of each factor was pre-set to 3. The corresponding level values of each factor are listed in Table 2. Refractive index, extinction coefficient, uniformity, and deposition rate were chosen as the response parameters. The orthogonal experiment table (Table 3) was designed by Orthogonality Design Assistant II v3.1 based on Table 2.

Range analysis and analysis of variance (ANOVA) are common methods used to analyze the response results of orthogonal experiments [34]. Range analysis is utilized to determine the degree of factors to response parameters and the optimal conditions. The calculation formulas are as follows:

(1) $$k_{i}=\frac{1}{n}\sum_{j=1}^{n}Y_{ij}$$

Here, *k_i_* is the mean value of level *i* (*i* = 1, 2, …). *n* (*n* = 1, 2, …) is the number of tests of level *i*. *j* (*j* = 1, 2, …) is the number of repetitions of each level. *Y_ij_* is the response value of level *i* with *j*-times repetition.

(2) $$Range\;value=\max\left(k_{i}\right)-\min(k_{i})$$

ANOVA is applied to analyze the contribution rates of factors to response parameters [35]. The calculation formulas are as follows:

(3) $$f_{K}=I_{K}-1$$

Here, *f_K_* is the degree of freedom (DOF) of the factor *K*.

(4) $$f_{tot}=N-1$$

Here, *f_tot_* is the total DOF. *N* is the number of orthogonal tests.

(5) $$f_{E}=f_{tot}-\sum_{k=1}^{K}f_{K}$$

Here, *f_E_* is the DOF of the error.

(6) $$T=\sum_{i=1}^{1}\sum_{j=1}^{J_{i}}Y_{ij}$$

Here, *T* is the sum of the indicator response values.

(7) $$Q=\sum_{i=1}^{1}\sum_{j=1}^{J_{i}}Y_{ij}^{2}$$

Here, *Q* is the square sum of the indicator response values.

(8) SSD=Q−T2N

Here, *SSD* is the total square sum of deviations.

(9) $${SS}_{K}=\sum_{i=1}^{I_{K}}\frac{{\left(\sum_{j=1}^{J_{i}}Y_{ij}\right)}^{2}}{J_{i}}-\frac{T^{2}}{N}$$

Here, *SS_K_* is the square sum of deviations of the factors.

(10) $${SS}_{E}=SSD-\sum_{k=1}^{K}{SS}_{K}$$

Here, *SS_E_* is the square sum of deviations of the error.

(11) $$P_{K}=100\%\times \frac{{SS}_{K}}{SSD}$$

Here, *P_K_* is the contribution rate of the factors.

In statistics, F-test is a widely used method to assess the significant difference between the variances of data sets. The calculation formula is as follows:

(12) $$F=\frac{SSD/f_{tot}}{{SS}_{E}/f_{E}}$$

Here, *F* is the F-ratio of the factors. In this study, the *f_tot_* = 8, the *f_K_* = 2, and the F-ratio of 95% confidence was selected according to the F-test critical value table [36], *F*_0.05_ = 4.46. If *F* ≥ *F*_0.05_, it was considered to have a significant impact; if *F* < *F*_0.05_, it was not significant.

### 2.3. Characterizations

Various characterization technologies were used to explore the structures of SiN_x_ thin films. Scanning electron microscope (Nova Nano SEM450, Waltham, MA, USA) was used to observe the surface morphology. Fourier-transform infrared (FTIR) (Thermo Scientific Nicolet iS10, Waltham, MA, USA) within the range of 400–4000 cm^−1^ was used for analyzing the functional groups. X-ray photoelectron spectroscopy (XPS) (Thermo Scientific K-Alpha, Waltham, MA, USA) could further analyze the element contents and bonding types.

### 2.4. Refractive Index, Extinction Coefficient, Uniformity, and Deposition Rate Tests

The refractive index, extinction coefficient and uniformity were tested via ellipsometer (full-spectrum ellipsometer SE95OFS-RD of Zhidong Optoelectronics Technology Co., Ltd., Shanghai, China) at an incidence angle of 60° with wavelength of 633 nm. The model of glass substrate and silicon nitride film was selected as the test fitting model. According to Fresnel’s formula, the properties of reflected light are directly dependent on the difference in refractive index between the incident medium and the reflecting medium, as well as on the incident angle of the light. Using the data obtained from the ellipsometer and the Fresnel equation, we could build a mathematical model to describe the reflection phenomenon. By solving this model in reverse, the refractive index, the extinction coefficient, and the thickness of the film could be deduced. Since the film was not repaired before the above-mentioned test, the area of 10 mm from the edge was not used as testing area. We took the center of the sample circle as the center point and the circle area with r = 30 mm was Zone 1. The ring area 30–60 mm from the center of the circle was Zone 2. The ring area 60–90 mm away from the center of the circle was Zone 3. Three test points were randomly selected in each Zone. But to avoid the distance between the selected points being too close, the distance difference between the two points and the center of the circle had to be ≥ 10mm, and the linear distance between the two points had to be ≥ 20mm. The film uniformity and the deposition rate (nm/s) were calculated using the following equation:

(13) $$uniformity\%=100\%\times \frac{d_{max}-d_{min}}{2\times \bar{d}}$$

Here, *d_max_*, *d_min_*, and $\bar{d}$ are the maximum, minimum and the average values of thickness of selected 3 points, respectively. The calculated values of $\bar{d}$ are listed in Table 4.

## 3. Results and Discussion

### 3.1. Statistical Analysis and Optimal Parameters

The orthogonal experimental analysis can be used to reveal the influence trend of the influence factors on the response parameters via the statistical method from the macro viewpoint.

**Figure 2:**
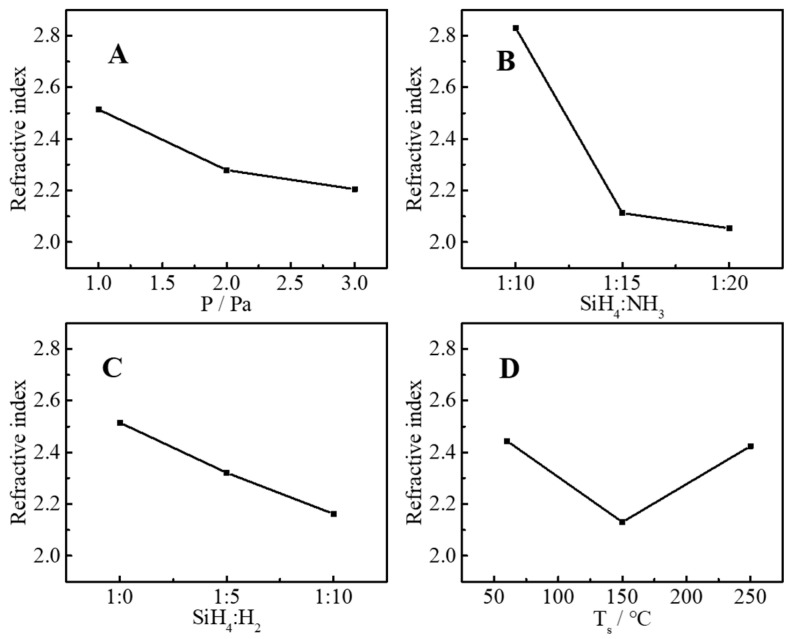
Influence of pressure (**A**), ratio of SiH_4_ to NH_3_ (**B**), ratio of SiH_4_ to H_2_ (**C**) and substrate temperature (**D**) on refractive index.

**Figure 3:**
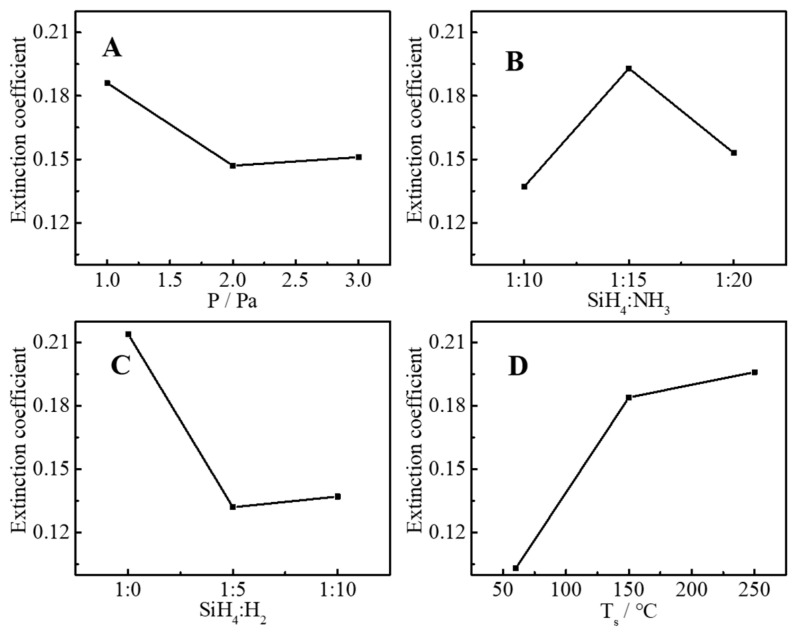
Influence of pressure (**A**), ratio of SiH_4_ to NH_3_ (**B**), ratio of SiH_4_ to H_2_ (**C**) and substrate temperature (**D**) on extinction coefficient.

**Figure 4:**
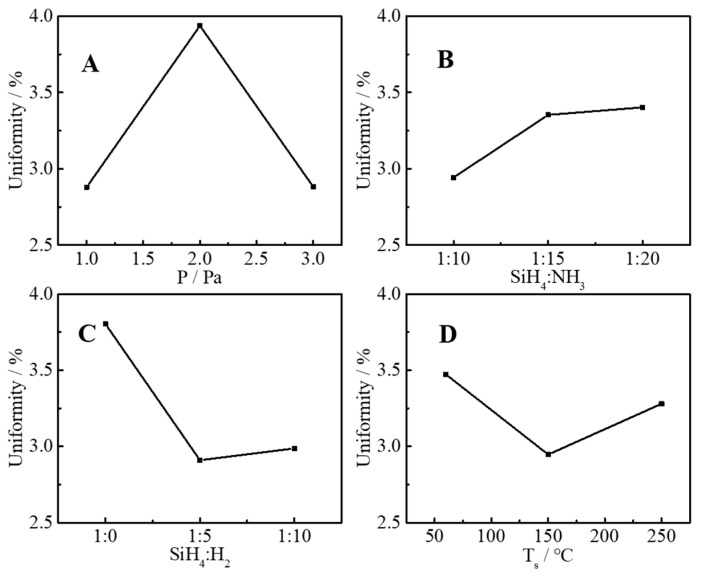
Influence of pressure (**A**), ratio of SiH_4_ to NH_3_ (**B**), ratio of SiH_4_ to H_2_ (**C**) and substrate temperature (**D**) on film uniformity.

**Figure 5:**
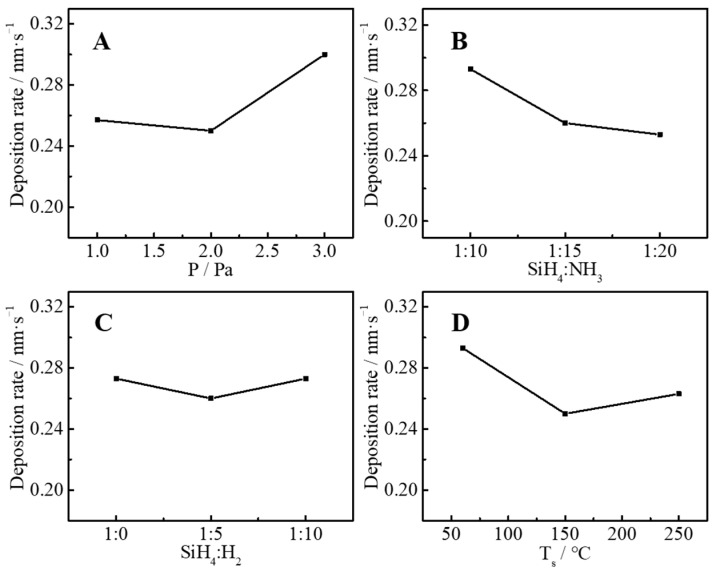
Influence of pressure (**A**), ratio of SiH_4_ to NH_3_ (**B**), ratio of SiH_4_ to H_2_ (**C**) and substrate temperature (**D**) on deposition rate.

According to the orthogonal experiment design table (Table 3), a total of nine groups of experiments were performed, and the refractive index, extinction coefficient, uniformity, and deposition rate of nine groups of samples were tested, and these four parameters were used to evaluating the film quality as response parameters. The test results are listed in Table 4. Figure 2, Figure 3, Figure 4 and Figure 5 are the main effect diagrams, and these display the influence trend of a single factor on different response parameters, and the optimal combination of factors for a single response parameter can be found. Table 5 calculates the range values of various conditions: the larger the range value is, the greater the influence is, and this can analyze the primary and secondary factors affecting the refractive index, extinction coefficient, uniformity, and deposition rate. It is not difficult to find that the main influencing factors of different response factors are different. For instance, the range values of the refractive index were SiH_4_:NH_3_ > SiH_4_:H_2_ > T_s_ > P, which suggested that the main influencing factor was SiH_4_:NH_3_ and the secondary influencing factor was SiH_4_:H_2_, and the sequence of influencing factors was as the same as that of range values. This may have been because SiH_4_, NH_3_, and H_2_ are the source gases for the preparation of silicon nitride films, and a change in their ratios directly affects the elemental composition and bonding of SiN_x_ thin films. The same method was used to judge the influence of the extinction coefficient, uniformity, and deposition rate, and the sequences of influencing factor were T_s_ > SiH_4_:H_2_ > SiH_4_:NH_3_ > P, P > SiH_4_:H_2_ > T_s_ > SiH_4_:NH_3_, and P > T_s_ > SiH_4_:NH_3_ > SiH_4_:H_2_, respectively. The influences of SiH_4_, NH_3_, and H_2_ were still important. But it can be suspected that T_s_ and P also caused changes in bonding types that affected the response parameters. The influence of elements and valence bonds on response parameters will be further discussed in the characterization section.

Analysis of variance (ANOVA) was used to statistically evaluate the significant differences in response parameters. The results are shown in Table 6. Since the F-value was not enough to achieve the condition of significant influence, the contribution rates of parameters were used to measure the influence of factors. The sequences of parameter contribution were consistent with range analysis. Since the refractive index, extinction coefficient, and uniformity can be analyzed by characterization technologies, the influencing conditions of the deposition rate are briefly discussed based on statistical analysis in this part. The most influential factor of the deposition rate is reaction pressure. High pressure would accelerate the pyrolysis of the source gases on the hot wire, which would make the free radicals more likely to combine with each other to form new bonds. Based on this condition, the substrate temperature affects bonding and nucleation growth as an important factor in film deposition.

In this work, the objective was to obtain a SiN_x_ thin film with a refractive index of 2.0. On this basis, the deposition rate of the film was as fast as possible, and the extinction coefficient and uniformity of the film were smaller, which was beneficial to its application in the field of anti-reflection films in solar cells. Following overall consideration, the preparation conditions of No. 9 were selected as the optimal parameters, and these reached the preparation of the ideal SiN_x_ quality in terms of the target refractive index (2.026), a low extinction coefficient (0.056), great uniformity (2.97%), and a fast deposition rate (0.3 nm/s).

### 3.2. Characterization Analysis

According to the statistical analysis results of the orthogonal experiment, SiH_4_:NH_3_, SiH_4_:H_2_, and T_s_ have large effects on the refractive index and extinction coefficient. So as to investigate the relationship between influence factors and different response parameters, several samples were selected for FTIR, XPS, and SEM characterization.

#### 3.2.1. FTIR

**Figure 6:**
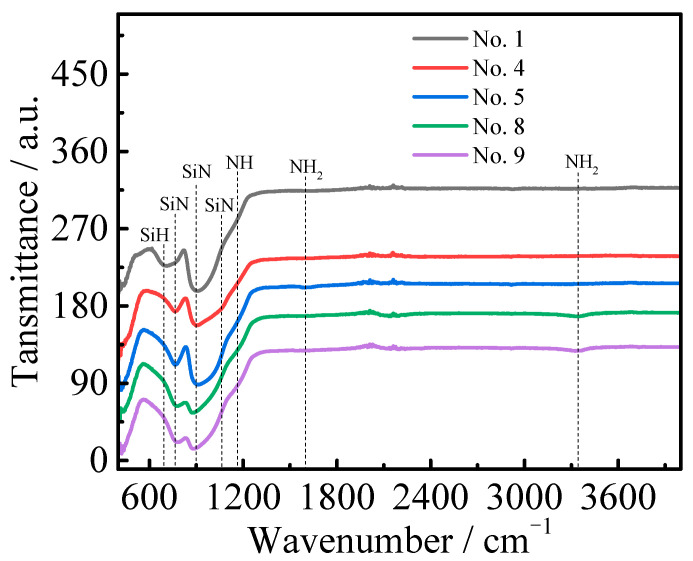
FTIR transmittance spectra of Nos. 1, 4, 5, 8, and 9.

Figure 6 displays the FITR transmittance spectra of Nos. 1, 4, 5, 8, and 9. It clearly shows the main peak at about 900 cm^−1^ due to the SiN asymmetric stretching mode and a shoulder at about 1180 cm^−1^, related to NH binding vibration [37,38]. Expressly, No. 1 had a broad peak at 700–800 cm^−1^, which was attributed to SiH wagging vibration (700 cm^−1^) and SiN symmetric stretching (~770 cm^−1^) [39]. The sharp peaks of Nos. 4, 5, 8, and 9 located at about 770 cm^−1^ and belonging to the SiN symmetric stretching mode had a slight tendency towards shifting toward a higher wavenumber as SiH_4_:NH_3_ increased. It was attributed to the induction effect facilitated by N atoms integrated into tetrahedral clusters with the increase in N content in SiN_x_ [38], which was also confirmed by the results for N content in XPS element analysis. According to the preparation process of each sample, Nos. 1 and 8, prepared without H_2_, and the formed SiN_x_ may have more suspension bonds. But No. 8 only had a peak with a SiN bond (~770 cm^−1^) without the SiH wagging vibration (700 cm^−1^), which could have been caused by increases in P and SiH_4_:NH_3_, the SiN bonding content was increased and a more stable H bond was formed. In Nos. 4, 5, and 9, H_2_ was added, effectively passivating a part of the suspension bond, bringing the overall structure of SiN_x_ to a stable state. No. 4 exhibited a shoulder at 1060 cm^−1^, which could have been caused by SiN asymmetric vibration; No. 5 showed a NH_2_ symmetric scissor vibration at 1600 cm^−1^; and No. 9 had a peak at 3350 cm^−1^ due to NH_2_ symmetric stretch vibration [39,40]. It can be speculated that NH_2_ of No. 5 was in an unstable state, which might have been due to the adsorption of the free radical formed via NH_3_ pyrolysis on the SiN_x_ surface. However, No. 4 had less H bond formation, which might have been owed to high T_s_ accelerating H escape [39].

#### 3.2.2. XPS

**Figure 7:**
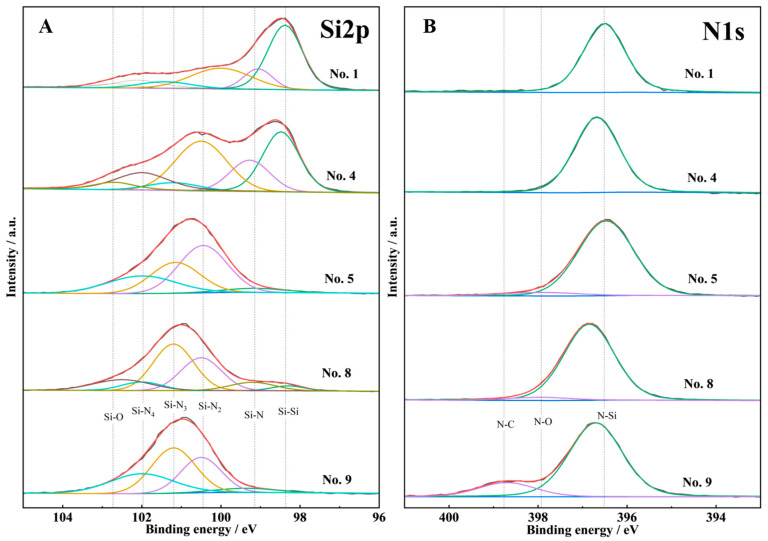
Deconvolution of Si2p (**A**) and N1s (**B**) XPS spectra of Nos. 1, 4, 5, 8, and 9.

Using the same samples that characterized FTIR, we also tested XPS spectra for analyzing the chemical environment of Si and N in SiN_x_ films. Since the ratios of source gases were different, the element ratio also had disparity. The result is exhibited in Table 7. In spite of the N content having increased significantly and the ratio of N/Si having risen from 0.17 (No. 1) to 0.75 (No. 9), the product still was a Si-rich SiN_x_ film rather than a standard stoichiometric Si_3_N_4_ (N/Si = 1.33). The deconvolution of N peaks was relatively simple (Figure 7B); it contained the main peak of Si-N at 396.5–396.8 eV and the shell peaks of N-O were located at 397.9 eV [41,42]. However, No. 9 deconvoluted a higher binding energy peak at 398.7 eV, which we attributed to N-C [43]. In contrast, Si2p spectra deconvolution was much more interesting. According to Figure 7A, the main peak of Si2p shifted to higher binding energy, and the shoulder peak gradually became the main peak from No. 1 to No. 9, indicating that the chemical environment of Si changed greatly. The Si2p peak could be deconvoluted into six peaks including those with Si-Si (98.4 eV), Si-N (99 eV), Si-N_2_ (100.5 eV), Si-N_3_ (101.2 eV), Si-N_4_ (102 eV), and Si-O (102.5 eV) [3,44,45]. The change in peak intensity implied a change in bonding content. In Nos. 1 and 4, the main peaks were Si-Si bonds, but the difference was that the Si-N_2_ content of No. 4 increased dramatically. It was worth noting that the refractive indexes of both Nos. 1 and 4 were relatively large. Hence, it could be assumed that an increase in the intensity of the Si-Si peak would lead to a rise in the refractive index, and the results of the comparison of Nos. 5, 8, and 9 were also consistent with this conclusion. However, the refractive indexes of Nos. 5 and 9 were close to 2.0; the difference only lay in the change in Si-N_2_ and Si-N_3_ contents caused by the decreased Si content [46]. The increase in the extinction coefficient was attributed to the Si content increasing, which was caused by the band gap decreasing [47]. Meanwhile, the sequence of extinction coefficient values was 8 > 1 > 4 > 5 > 9, which was also consistent with the sequence of Si content; except No. 8 may have been affected by the content of the Si-O bond to some extent.

#### 3.2.3. SEM

**Figure 8:**
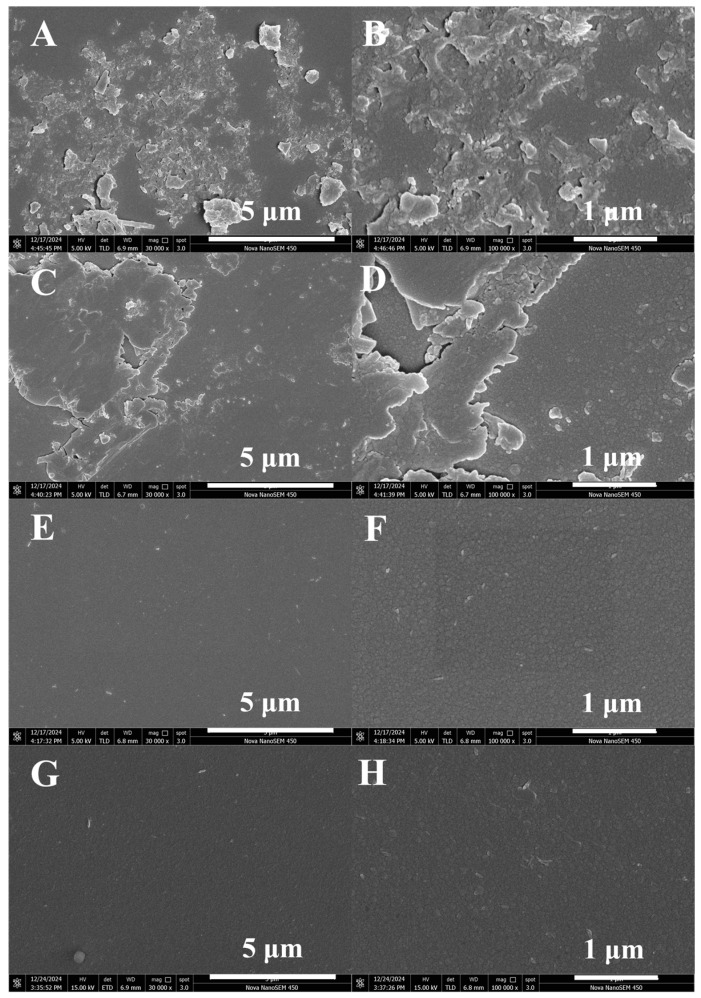
SEM images of SiN_x_ surfaces of Nos. 1 (**A**,**B**), 6 (**C**,**D**), 7 (**E**,**F**), and 9 (**G**,**H**).

The uniformity can be visually observed from the surface morphology images (Figure 8). Considering that the reaction pressure was the largest influence factor, four samples were chosen to observe the surface microstructures, which had the maximum uniformity (No. 6), the minimum uniformity (No. 7), a reaction pressure of 1 Pa (No. 1), and the optimal parameter (No. 9). Nos. 1 and 6 showed irregular surface bulges, and even formed some SiN_x_ chunks locally on the film surface, indicating that SiN_x_ had been deposited unevenly on the glass substrate surface, which was in sharp contrast to the flat surfaces of Nos. 7 and 9. At a rough glance, both Nos. 7 (E) and 9 (G) had flat surfaces. But after magnification, No. 9 (H) had some more obvious irregular island structures than No. 7 (F). The islands were not completely fused to a flat film, and there existed obvious gaps, which may have contributed to the poorer uniformity of No. 9.

## 4. Conclusions

This study successfully optimized the preparation process of high-uniformity SiN_x_ thin films with a target refractive index using Cat-CVD through the orthogonal experiment design method. The optimal preparation conditions, including P = 3 Pa, SiH_4_:NH_3_ = 1:20, SiH_4_:H_2_ = 1:5, and T_s_ = 60 °C, produced SiN_x_ thin films with a refractive index of 2.026, an extinction coefficient of 0.056, a uniformity of 2.97%, and a deposition rate of 0.3 nm/s. The effect tendency and importance of each factor affecting the response parameters was analyzed by statistical methods. The results demonstrated that the ratio of SiH_4_:NH_3_ and SiH_4_:H_2_ were the primary factors influencing the refractive index and extinction coefficient in SiN_x_ films. P and T_s_ altered the bonding mode of SiN_x_, influencing film surface uniformity. These conditions finally led to different elemental compositions and structures, thereby affecting the refractive index and extinction coefficient. Characterization analysis using SEM, FTIR, and XPS discussed the microstructure property relationships of SiN_x_ thin films under various preparation conditions. It was revealed that the differences in element contents and bonding types were the most fundamental reasons affecting the properties of SiN_x_ thin films. This study indicated that the Cat-CVD is a promising method for the preparation of SiN_x_ thin films with controllable properties and laid a foundation for the further development of SiN_x_ films suitable for different scenarios and investigation of the mechanism of the influence of factors on the properties of these films.

## Figures and Tables

**Table 2:** Values of each level for 4 factors.

Level	Experimental Factor			
	P/Pa	SiH_4_:NH_3_	SiH_4_:H_2_	T_s_/°C
1	1	1:10	1:0	60
2	2	1:15	1:5	150
3	3	1:20	1:10	250

**Table 3:** Orthogonal experimental design *L*_9_(3)^4^.

No.	Experimental Factor			
	P/Pa	SiH_4_:NH_3_	SiH_4_:H_2_	T_s_/°C
1	1	1:10	1:0	60
2	1	1:15	1:5	150
3	1	1:20	1:10	250
4	2	1:10	1:5	250
5	2	1:15	1:10	60
6	2	1:20	1:0	150
7	3	1:10	1:10	150
8	3	1:15	1:0	250
9	3	1:20	1:5	60

**Table 4:** Response results of orthogonal experiment.

No.	Response Parameter				
	Refractive Index	Extinction Coefficient	$\bar{d}\;(\pm ∆d)$/nm	Uniformity/%	Deposition Rate/nm·s^−1^
1	3.306	0.157	149.440 (±5.081)	3.40	0.31
2	2.079	0.212	105.177 (±2.514)	2.39	0.22
3	2.157	0.189	177.222 (±5.051)	2.85	0.24
4	2.857	0.129	125.333 (±4.224)	3.37	0.26
5	1.999	0.097	127.888 (±5.179)	4.05	0.27
6	1.980	0.215	103.270 (±4.534)	4.39	0.22
7	2.330	0.126	151.030 (±3.111)	2.06	0.31
8	2.259	0.271	138.220 (±5.004)	3.62	0.29
9	2.026	0.056	144.000 (±4.277)	2.97	0.30

**Table 5:** The range analysis of various response parameters.

Response Parameter	Factor	Mean Value			Range Value
		k_1_	k_2_	k_3_	
Refractive index	P	2.514	2.279	2.205	0.309
	SiH_4_:NH_3_	2.831	2.112	2.054	0.777
	SiH_4_:H_2_	2.515	2.321	2.162	0.353
	T_s_	2.444	2.130	2.424	0.314
Extinction coefficient	P	0.186	0.147	0.151	0.039
	SiH_4_:NH_3_	0.137	0.193	0.153	0.056
	SiH_4_:H_2_	0.214	0.132	0.137	0.082
	T_s_	0.103	0.184	0.196	0.093
Uniformity/%	P	2.880	3.937	2.883	1.057
	SiH_4_:NH_3_	2.943	3.353	3.403	0.460
	SiH_4_:H_2_	3.803	2.910	2.987	0.893
	T_s_	3.473	2.947	3.280	0.526
Deposition rate/nm·s^−1^	P	0.257	0.250	0.300	0.050
	SiH_4_:NH_3_	0.293	0.260	0.253	0.040
	SiH_4_:H_2_	0.273	0.260	0.273	0.013
	T_s_	0.293	0.250	0.263	0.043

**Table 6:** The ANOVA of various response parameters.

Response Parameter	Factors	SSD	P_K_/%	F
Refractive index	P	0.156	9.43	0.377
	SiH_4_:NH_3_	1.123	67.94	2.717
	SiH_4_:H_2_	0.188	11.38	0.455
	T_s_	0.186	11.25	0.450
Extinction coefficient	P	0.003	8.33	0.333
	SiH_4_:NH_3_	0.005	13.89	0.556
	SiH_4_:H_2_	0.013	36.11	1.444
	T_s_	0.015	41.67	1.667
Uniformity/%	P	2.226	49.41	1.976
	SiH_4_:NH_3_	0.382	8.48	0.339
	SiH_4_:H_2_	1.471	32.65	1.306
	T_s_	0.426	9.46	0.378
Deposition rate/nm·s^−1^	P	0.004	40.00	1.600
	SiH_4_:NH_3_	0.003	30.00	1.200
	SiH_4_:H_2_	0.000	0.00	0.000
	T_s_	0.003	30.00	1.200

**Table 7:** Silicon and nitrogen element contents and ratio of N to Si in SiN_x_ films.

No.	Element Content %		N/Si
	Si	N	
1	85.67	14.33	0.17
4	74.98	25.02	0.33
5	61.73	38.27	0.62
8	57.38	42.62	0.74
9	57.07	42.93	0.75

## Data Availability

Data are contained within the article.
